# Enhancing OSCE reliability and effectiveness in radiology resident training with long-term systemic evaluation

**DOI:** 10.1186/s13244-025-02024-3

**Published:** 2025-09-23

**Authors:** Ning Ding, Xinyu Gao, Hao Sun, Lan Song, Xuan Wang, Yu Chen, Daming Zhang, Zhengyu Jin, Huadan Xue

**Affiliations:** Department of Radiology, Peking Union Medical College Hospital, Chinese Academy of Medical Sciences and Peking Union Medical College, Beijing, China

**Keywords:** Objective structured clinical examination (OSCE), Radiology resident training, Examination scores, Difficulty index, Clinical competence

## Abstract

**Objective:**

This study aimed to evaluate the long-term systematic effectiveness and reliability of the Objective Structured Clinical Examination (OSCE) in radiology resident training, from the perspectives of both examiners and examinees.

**Methods:**

This retrospective observational study analyzed subjective evaluations and objective examination data collected over 6 years (2018–2021, 2023, and 2024). Subjective evaluations were gathered via questionnaires from 198 examiners and 818 examinees to assess the difficulty and satisfaction with the OSCE. Objective data, including examination scores, difficulty indices, and discrimination indices, for each OSCE station were analyzed using correlation analysis and *t*-tests.

**Results:**

The OSCE demonstrated stable performance over 6 years, with consistent difficulty levels and discrimination ability across all stations. The average scores for individual stations varied; however, the overall final scores remained stable. Strong correlations between the station and final scores indicate good discrimination. Examinees rated the overall difficulty higher than examiners, but the objective indices aligned with examiner assessments. Over 6 years (198 examiners, 818 examinees), OSCE scores stabilized (85.48–88.48), with improved consistency (station range narrowed to 85.51–93.9 by 2024). Difficulty (0.12–0.15) and discrimination indices remained stable (most *p* < 0.05). Examinees rated it harder than examiners (*p* < 0.001).

**Conclusion:**

The OSCE is a reliable, valid, and effective assessment tool in radiology. Evaluating the OSCE from both subjective and objective perspectives ensured the robustness and validity of the examination.

**Critical relevance statement:**

This 6-year study evaluates the Objective Structured Clinical Examination (OSCE) in radiology training through multidimensional analysis of examination metrics (difficulty indices and discrimination coefficients) and stakeholder feedback (*n* = 198 examiners, 818 examinees), demonstrating its consistency for clinical competency assessment.

**Key Points:**

- The radiology OSCE demonstrated consistent reliability, stable difficulty indices, and strong score correlations.
- Examinees overestimated exam difficulty compared to examiners, likely due to stress-related perception bias.
- Standardized examiner training improved scoring consistency and enhanced overall OSCE assessment quality.

**Graphical Abstract:**

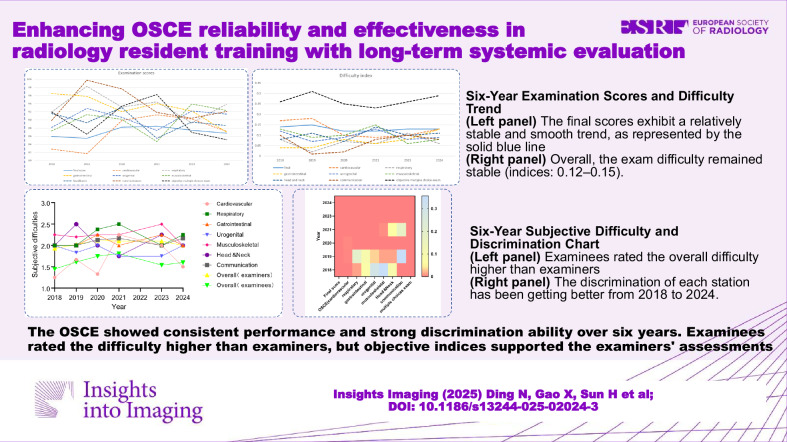

## Introduction

The Objective Structured Clinical Examination (OSCE) is a performance-based assessment widely used in medical education to evaluate residents’ clinical skills. The OSCE consists of a series of stations dedicated to specific clinical tasks or competencies. Residents progress through these stations, typically allocating 5–10 min per station to perform the designated tasks, while examiners observe and assess their performance against predefined criteria. Since its inception in 1975 at the University of Dundee, Scotland, the OSCE has been a pivotal tool in medical education. Developed by Barrows and Norman, the OSCE was designed in response to the need for a more structured and objective method of evaluating the clinical competencies of medical students [1]. Over the years, the OSCE has been globally adopted across various medical disciplines as a standardized method for assessing clinical competence in undergraduate education, postgraduate training, and licensing examinations [2–5]. In China, the OSCE was introduced in radiology resident training in 2014. To implement this new examination format, examination organizers place significant emphasis on OSCE evaluations from both examiner and examinee perspectives, as well as on examiner training [6].

A comprehensive evaluation of the OSCE necessitates considering the perspectives of both examinees and examiners [7–9]. One of the primary objectives of medical education assessment is to minimize the factors that could influence test outcomes, striving to generate scores that accurately reflect a learner’s true abilities with maximal reliability and validity. Consequently, an objective analysis of examination scores is crucial for assessing the effectiveness and reliability of the OSCE, employing methodologies such as the difficulty index and discrimination index, as described by Tavakol et al [10]. In addition to the evaluation of examinations, effective examiner training is essential to standardize assessments and minimize bias, thereby ensuring accurate scoring. The key components of this training include a thorough understanding of the OSCE objectives, calibration, standardization, training in scoring rubrics, and awareness of potential bias [11, 12].

This study aimed to conduct a comprehensive 6-year evaluation of the OSCE system used in radiology resident training in Beijing, China. It assesses the effectiveness, reliability, and validity of the OSCE from both examiner and examinee perspectives by utilizing subjective evaluations through questionnaires and objective data such as examination scores, difficulty indices, and discrimination indices.

## Methods

This retrospective observational study analyzed OSCE data from a single academic medical center (Peking Union Medical College Hospital) over six non-consecutive years (2018–2021, 2023–2024), excluding 2022 due to COVID-19 restrictions, which necessitated a fully online format with multiple-choice questions and text-based responses on a computer, compromising assessment standardization.

### Participants

A total of 198 examiners and 818 examinees participated in the OSCE examination for radiology residents from 20 hospitals in Beijing, China. The examinees ranged in age from 27.46 to 28.20 years, encompassing various educational degrees. As this retrospective study analyzed population-level assessment data, effect sizes rather than power calculations drive interpretation.

### OSCE setting

The OSCE comprises seven stations, six of which focus on different clinical systems—cardiovascular, respiratory, gastrointestinal, urogenital, musculoskeletal, and head and neck—and one dedicated to communication skills. Each station was allocated 9 min, with an additional minute designated for transitioning between stations.

The OSCE questions were developed by the same group of question developers throughout the 6-year examination period, with each question developer responsible for two OSCE stations. Benchmarking sessions, involving a review of performance data from previous years’ OSCE stations, were conducted prior to question development to ensure consistency and quality.

### Examiner selection and training

To standardize the evaluation process, a pre-examination training session was conducted with the examiners. This training aimed to familiarize them with the competencies being assessed, including clinical skills, communication, and decision-making. The examiners received a sample test and score sheet to promote consistency in scoring. Calibration sessions were conducted to align the examiners’ scores with established rubrics, thereby reducing variability and ensuring interrater reliability. Additionally, to minimize bias, the examiners were trained to observe behaviors objectively and avoid overreliance on subjective impressions. All examiners held the title of Associate Professor or above, as mandated by China’s National Health Commission (NHC) for physician qualification assessments. This criterion ensures adherence to national standards of clinical and teaching competency, as NHC-certified titles require rigorous evaluations of medical expertise, research output, and educational experience.

### Evaluation methods

Both subjective and objective evaluations were employed to assess the OSCE’s effectiveness.

#### Subjective evaluation

Both examiners and examinees were invited to anonymously complete a self-administered questionnaire via the electronic platform WJX.cn, which collected demographic information and subjective assessments of examination difficulties. Examinees evaluated the overall difficulty of the exam using a three-point Likert scale (1 = “very difficult,” 2 = “moderate,” and 3 = “not difficult”). The examiners assessed the difficulty of their assigned stations using the same scale. Additionally, examiners provided feedback on the pre-OSCE training sessions, evaluating their satisfaction on a three-point Likert scale (1 = “very satisfied,” 2 = “moderate,” and 3 = “not satisfied”). The examinees are not obligated to complete the questionnaire, and their responses have no impact on their final examination scores. A three-point Likert scale in our study was used with careful consideration of both methodological efficiency [13] and the practical constraints [14] of our survey context.

#### Objective evaluation

Objective evaluations included the calculation of average scores, difficulty indices, discrimination indices for each OSCE station, and the overall examination. The difficulty index was determined by dividing the mean score of all the candidates by the total test score. The discrimination index was assessed using the extreme group method, with *p*-values < 0.05, indicating a good discrimination ability.

### Statistical analysis

Statistical analyses were conducted using Statistical Package for the Social Sciences (SPSS) Statistics software (version 21.0, IBM). Descriptive statistics, including means and standard deviations, were calculated for all variables. To evaluate differences across years, *t*-tests, Mann–Whitney tests, and Kruskal–Wallis tests were used. The final performance score for each examinee was calculated as the average of the two examiners’ scores. Correlation analyses were performed to assess the relationship between each OSCE station’s score and the final score.

### Ethical considerations

This study was approved by the Institutional Review Board of the conducting institution (No. S-K2067). All participants provided written informed consent prior to inclusion in the study.

## Results

### Questionnaire evaluation

In total, 198 and 559 questionnaires were collected, respectively (Table 1). The examiner and examinee response rates were 100% and 68.37%, respectively. The demographic characteristics of the examiners and examinees are shown in Table 1.

**Table 1:** The population information of examiners and examinees

Examiner							
Year	Age (years)	Gender		Position			
		Male	Female	Associate professor		Professor	
2018	40.44 ± 4.032	10	19	26		3	
2019	41.93 ± 4.509	12	34	39		7	
2020	42.86 ± 4.837	10	29	32		7	
2021	44.36 ± 4.434	9	20	23		6	
2023	45.07 ± 4.537	11	19	20		10	
2024	46.25 ± 5.261	5	20	15		10	

Examinee							
Year	Age (years)	Gender		Degree			
		Male	Female	Bachelor	Master		Doctor
2018	27.71 ± 1.916	24	63	17	53		17
2019	28.05 ± 2.311	38	88	32	71		23
2020	28.18 ± 2.352	19	53	27	36		9
2021	27.93 ± 2.540	28	61	29	51		9
2023	27.46 ± 2.252	24	60	34	44		6
2024	28.20 ± 3.033	34	67	35	56		10

**Fig. 1:**
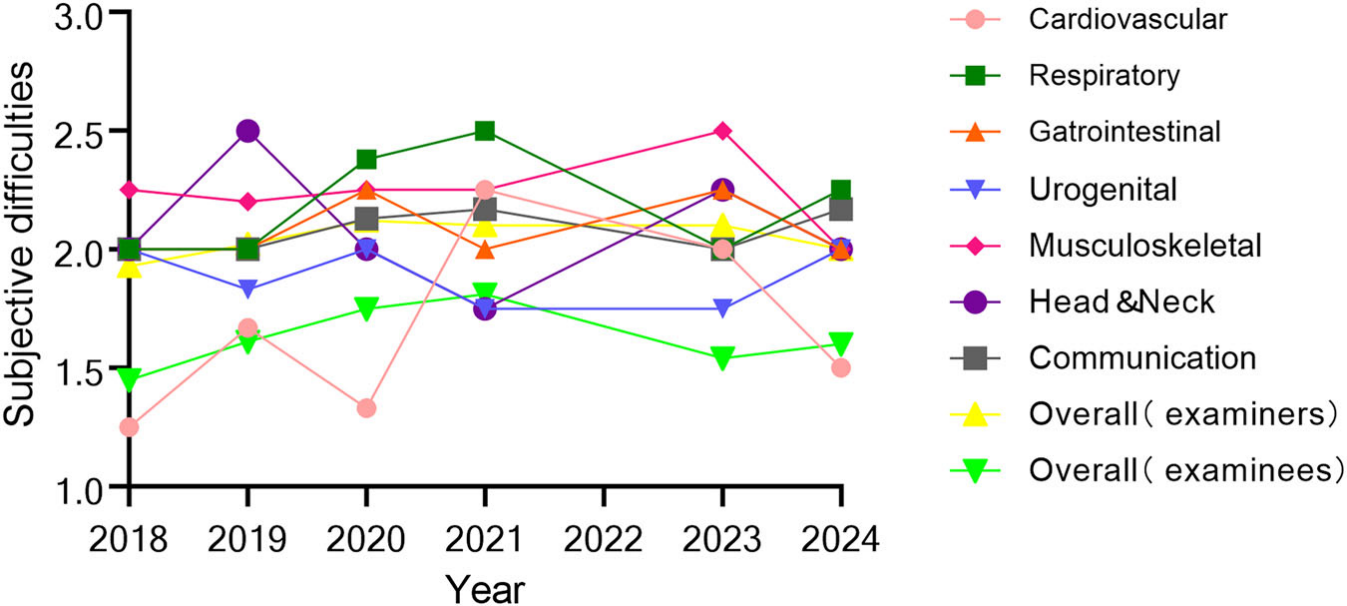
The subjective difficulties rating scores of examiners and examinees. Examinees rated the overall difficulty higher than examiners, but the objective indices aligned with examiner assessments

The subjective difficulty ratings for the OSCE, as reported by both examiners and examinees, are presented in Fig. 1 (and Supplemental Table 1). An analysis of the examiners’ subjective difficulty ratings across various systems revealed no significant differences over time. Independent *t*-tests for each system, including the cardiovascular (*p* = 0.098), respiratory (*p* = 0.213), gastrointestinal (*p* = 0.540), urogenital (*p* = 0.697), musculoskeletal (*p* = 0.862), head and neck (*p* = 0.130), and communication (*p* = 0.610) systems, did not show significant annual variations. Furthermore, there was no significant difference in the overall difficulty ratings across all OSCE stations over the years (*p* = 0.404).

A Kruskal-Wallis test for multiple independent samples was conducted to assess examiners’ subjective satisfaction with pre-OSCE training over the years (Table 2). The results showed no significant difference (*p* = 0.18). Post hoc pairwise comparisons showed that there were significant differences in satisfaction in 2020 (*p* = 0.023), 2021 (*p* = 0.021), and 2024 (*p* = 0.009) compared to 2018.

**Table 2:** Pre-OSCE examiner training subjective evaluation

Year	2018	2019	2020	2021	2023	2024
Satisfaction	1.24 ± 0.44	1.09 ± 0.29	1.05 ± 0.22*	1.07 ± 0.26	1.03 ± 0.18*	1.00 ± 0.00*

* Post hoc pairwise comparisons showed significant differences compared to 2018

A significant difference in the subjective difficulty rating scores for the overall OSCE stations was observed among the examinees between 2018 and 2020 (*p* = 0.002) and between 2018 and 2021 (*p* < 0.001). However, no significant differences were found in the remaining years. The subjective difficulty rating score was significantly different between examinees and examiners (*p* = 0.000), with examinees experiencing difficulty (mean rank = 342.43) and examiners feeling moderate difficulty (mean rank = 482.26).

### Examination score analysis

The statistical results for the scores of each OSCE station, objective multiple-choice examinations, final scores, correlations between each OSCE station and the average final score, and the difficulty and discrimination indices are presented below.

**Fig. 2:**
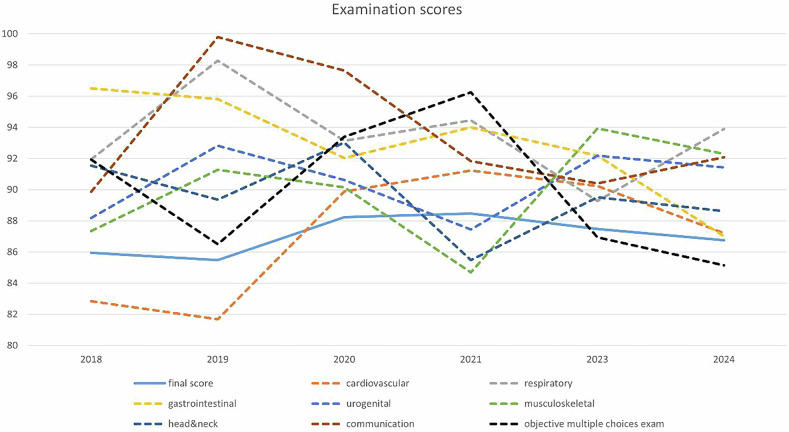
Line chart of examination scores over 6 years. The chart shows the final score (solid blue line) and scores for individual OSCE stations (gastrointestinal, head and neck, cardiovascular, urogenital, communication, respiratory, musculoskeletal) and the objective multiple-choice exam from 2018 to 2024. Final scores remained stable, with the highest in 2021 (88.48) and the lowest in 2019 (85.48)

Significant differences were observed in individual OSCE station scores, objective multiple-choice questions, and final scores across the 6 years. Longitudinal trends in Fig. 2 demonstrate a reduction in variability between the highest and lowest station OSCE scores, narrowing from 81.69–99.8 in 2019 to 85.51–93.9 in 2024.

Correlation analysis of the average total scores and the average scores for each station over 6 years revealed that all stations demonstrated correlation coefficients greater than 0.4, with all *p* < 0.05, indicating moderate and statistically significant correlations except for the communication station. Notably, the multiple-choice examination exhibited a strong correlation with the total score (r = 0.872), indicating excellent correlations (Table 3).

**Table 3:** Correlation coefficient of each OSCE station compared with the final score

Examination score over 6 years	Average score	Correlation coefficient	*p*-value
Final score	86.76 ± 5.66	/	/
OSCE station 1 (Cardiovascular)	80.00 ± 32.80	0.438	0.000
OSCE station 2 (Respiratory)	73.31 ± 34.11	0.569	0.000
OSCE station 3 (Gastrointestinal)	78.59 ± 33.77	0.445	0.000
OSCE station 4 (Urogenital)	74.28 ± 35.39	0.522	0.000
OSCE station 5 (Musculoskeletal)	75.22 ± 34.00	0.424	0.000
OSCE station 6 (Head and Neck)	75.16 ± 34.07	0.497	0.000
OSCE station 7 (Communication)	87.14 ± 14.31	0.197	0.000

**Fig. 3:**
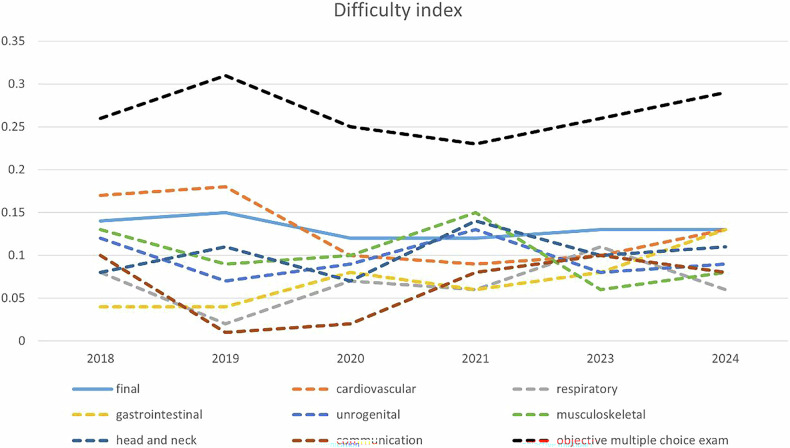
Line chart of difficulty indices over 6 years. The figure displays the difficulty indices for the final score (solid line), individual OSCE stations, and the objective multiple-choice exam from 2018 to 2024. Difficulty indices ranged from 0.12 to 0.15, with the cardiovascular station being the most difficult in 2019 (0.18) and the communication station the least (0.01). The multiple-choice exam peaked in difficulty in 2019 (0.31)

The trends in difficulty indices for each OSCE station, objective multiple-choice examination, and the final score over the 6 years were similar to those of the average scores (Fig. 3). Overall, the difficulty of the examination remained relatively stable, with difficulty indices ranging from 0.12 to 0.15. However, the difficulty indices for the objective multiple-choice examination and each OSCE station varied significantly across years. For the objective multiple-choice examination, the minimum difficulty index observed in 2021 is 0.23, and the maximum difficulty index was 0.31 in 2019. In the OSCE station, the minimum difficulty coefficient was observed for the communication station (0.01, 2019), whereas the maximum difficulty coefficient was recorded for the cardiovascular system station (0.18, 2019). The range between the lowest and highest difficult indices was narrowed down from 0.01–0.18 in 2019 to 0.06–0.13 in both 2023 and 2024.

**Fig. 4:**
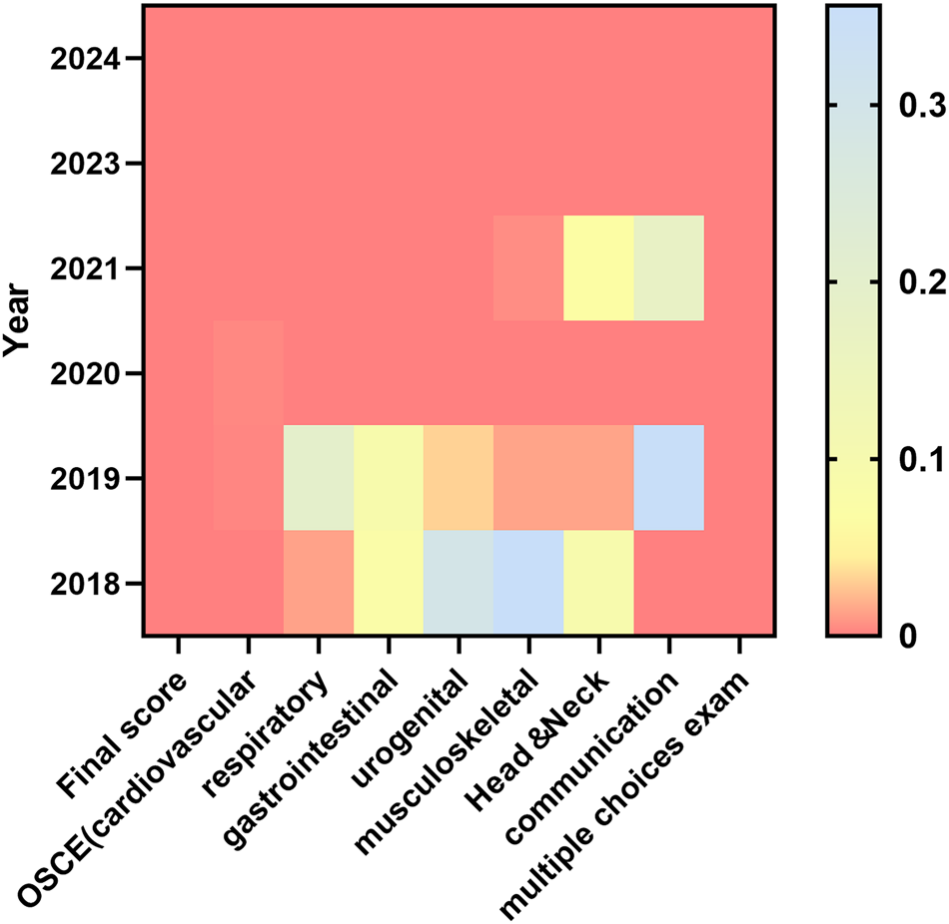
Discrimination of the final scores and individual OSCE station for 6 years. An OSCE station discrimination *p*-value less than 0.05 indicates that the station has good discrimination. In the color bar chart, yellow represents a *p*-value of 0.05. The figure illustrates a clear trend of improving station discrimination from 2018 to 2024

The discrimination ability of the examination remained generally good over 6 years (*p* < 0.05) (Fig. 4, Supplemental Table 2). However, in 2018, 2019, and 2021, some OSCE stations had *p* ≥ 0.05, including the gastrointestinal, urogenital, musculoskeletal, and head and neck stations in 2018, the respiratory, gastrointestinal, and communication stations in 2019, and the head and neck and communication stations in 2021.

## Discussion

### Evaluation of OSCE and improvement of its stability

Analysis of the OSCE over a 6-year period demonstrates the improvement of exam stability caused by the whole evaluation of the exam and feedback to question developers.

The overall stability of the final scores ranged from 85.48 in 2019 to 88.48 in 2021. Although significant differences were observed in final scores across the 6 years, there is only 3 points score difference between the highest and the lowest final scores. Longitudinal trend analysis demonstrate a reduction in variability between the highest and lowest OSCE station scores, narrowing from 81.69–99.8 in 2019 to 85.51–93.9 in 2024, indicating enhanced stability and consistency in the assessment outcomes.

### For the difficulty index of OSCE, a similar trend of increased stability was observed

Over the 6-year period, the overall difficulty index of the examination remained relatively stable, ranging from 0.12 to 0.15. However, notable variability existed in individual OSCE station difficulty indices in earlier years. For instance, in 2019, the difficulty indices ranged widely from 0.01 to 0.18, reflecting inconsistency across stations. By contrast, in both 2023 and 2024, the range had narrowed considerably to 0.06–0.13, indicating a more uniform level of difficulty across stations. This convergence suggests that the implementation of comprehensive examination evaluation and the regular feedback provided to item writers contributed to a more standardized and balanced assessment framework. The observed improvements highlight the importance of ongoing quality assurance practices in high-stakes clinical assessments. The design, monitoring, and feedback processes are crucial in enhancing the reliability and validity of OSCEs by reducing construct-irrelevant variance and improving item performance consistency [4].

### Effectiveness of OSCE as an assessment tool

The effectiveness of the OSCE is supported by its demonstrated discriminatory capacity at both the overall and station levels. Across the 6-year period, the final scores consistently exhibited high discriminatory power, with all t-values being statistically significant (*p* < 0.001), indicating a strong ability to distinguish between high- and low-performing examinees. However, we acknowledge that the discrimination ability of individual OSCE stations varied across years. In particular, several stations did not reach statistical significance in specific years—four stations in 2018, three in 2019, and two in 2021 (*p* ≥ 0.05)—suggesting a lack of sufficient evidence for discrimination in those cases. This limitation may be attributed to variation in station design, examiner inconsistency, or insufficient station sensitivity. Importantly, a trend toward improved station-level performance was observed in recent years: by 2023 and 2024, all OSCE stations achieved statistically significant discriminatory power. This improvement highlights the impact of continuous evaluation, item refinement, and feedback to station developers, which collectively contributed to enhanced validity and consistency in the assessment framework.

These findings highlight that the evaluation of OSCE performance can improve its stability and effectiveness. Through its multi-station design, each focusing on specific clinical competencies, the OSCE provides a comprehensive assessment of the candidates’ abilities in real-world clinical scenarios [15]. The significant differences observed in station-specific scores highlight the varied challenges posed by different clinical tasks. This aligns with the foundational role of OSCEs in medical training, where they not only assess technical skills but also evaluate critical thinking, communication, interaction and the ability to apply knowledge under pressure [16]. The comprehensive evaluation of examination scores, difficulty indices, and discrimination ability over the 6-year period demonstrated a marked improvement in the stability and effectiveness of the OSCE as an assessment method, which is consistent with previous studies [17].

### Perspectives from examiner and examinee

The use of questionnaires by both examiners and examinees is a crucial method for assessing the effectiveness of the OSCE. Collecting feedback from both groups ensured that the questions in the OSCE met the needs of evaluating the multiple competences of the residents [18, 19]. By surveying the examiners who evaluated the students at each OSCE station, we identified the questions at different OSCE stations in detail. The results of the study revealed that while the objective difficulty ratings from examiners varied across different OSCE stations and years, the overall difficulty remained stable over the 6-year period. This proves that the question design of the OSCE was highly qualified. As indicated by previous studies, examiner feedback is necessary to improve scoring consistency and evaluate the examination design [20]. The value of the evaluation from the examinees was considered equivalent to that provided by the examiners in the context of the OSCE [21]. In this study, the examinees’ subjective difficulty of the OSCE was higher than that reported by the examiners. However, when combined with the objective difficulty index of the examination scores, the real difficulty was close to the subjective difficulty of the examiners. In this study, the subjective difficulty reported by examinees for the OSCE was higher than that reported by examiners. However, when combined with the objective difficulty index derived from the examination scores, the actual difficulty closely aligns with the subjective difficulty, as assessed by the examiners. This discrepancy may be attributed to the stressful nature of the OSCE for residents, which likely increases their perception of the difficulty of the questions [22, 23].

### Importance of examiner training

Examiner training plays a significant role in minimizing scoring biases, such as leniency or severity, and enhancing the reliability of assessments [24, 25]. Our findings also emphasize the crucial role of examiner training in ensuring the objectivity and fairness of the OSCE. The consistent results across years, coupled with the pre-examination training provided to the examiners, underscore the importance of standardized training in calibration and scoring rubrics. The results of the questionnaire evaluation showed that examiner satisfaction with training varied with some notable differences between the years, particularly in 2020, 2021, and 2024. The rating scores for 2020, 2021, and 2024 indicate an improvement in satisfaction with pre-examination training compared to the previous 3 years, which may be attributed to accumulated experience. This variation indicates that a continuous and periodic review of examiner training programs is essential for maintaining high-quality evaluation standards [11]. By aligning the evaluators’ understanding and application of the assessment criteria, examiner training ensures greater consistency and fairness, which are vital for maintaining the validity and reliability of the OSCE.

### Study limitations and recommendations for future improvements

This study had certain limitations. First, the retrospective design inherently limits causal inferences, though our 6-year dataset provides robust observational evidence. Second, while the 3-point Likert scale (difficult/moderate/easy) was chosen to maximize response rates during time-sensitive post-exam evaluations, future studies could adopt a 5-point scale for more nuanced feedback [13, 14]. Third, while we excluded 2022 online OSCE data to maintain consistency, the pandemic’s impact on training may have affected 2021–2023 results; ongoing analysis of virtual/hybrid OSCE formats will help address this challenge moving forward. Last, the inconsistent performance across stations and weaker correlation coefficients at communication stations suggest the need for further investigation into the consistency of the assessment criteria.

To address these limitations, future prospective studies should focus on enhancing the objectivity and reliability of the OSCE. Implementing more structured and standardized feedback from both examiners and examinees could provide deeper insights into the station design and assessment criteria. Additionally, integrating advanced technologies such as AI-driven simulators may improve evaluation consistency and objectivity. These advancements can enhance the assessment process and contribute to the overall quality of radiology resident training.

## Conclusion

In conclusion, this study confirmed that the OSCE assessment system in radiology is a reliable, valid, and effective tool for evaluating clinical competencies. The stability of the difficulty indices and consistent discrimination ability across all stations further reinforced the robustness of the OSCE as a comprehensive assessment method. The integration of subjective evaluations from both examiners and examinees, along with the objective analysis of examination scores, underscores the importance of a multidimensional approach to ensure the examination’s objectivity and effectiveness.

Continuous improvements in examiner training programs are essential for maintaining high standards of assessment reliability and fairness. By leveraging these insights, the OSCE can strengthen its role in developing competent and well-rounded radiologists, ultimately contributing to improved patient care.

## Supplementary information

ELECTRONIC SUPPLEMENTARY MATERIAL

## Data Availability

The datasets used and/or analyzed during the current study are available from the corresponding author on reasonable request.

## Abbreviations

OSCE: Objective structured clinical examination
