# Supplementary material for: Enhancing OSCE reliability and effectiveness in radiology resident training with long-term systemic evaluation
Source: Insights Imaging. 2025 Sep 23;16:203. doi: 10.1186/s13244-025-02024-3 (PMC12457269; doi:10.1186/s13244-025-02024-3)
Supplement: Supplementary file 1 — ELECTRONIC SUPPLEMENTARY MATERIAL [file 13244_2025_2024_MOESM1_ESM.pdf]

Enhancing OSCE Reliability and Effectiveness in Radiology Resident Training with Long-Term Systemic Evaluation

ELECTRONIC SUPPLEMENTARY MATERIAL

Insights Imaging (2025) Ding N, Gao X, Sun H, et al.

Supplemental Table 1. The subjective difficulties rating scores of examiners and examinees.

Year	2018	2019	2020	2021	2023	2024
**Examiner Subjective Difficulty**						
**OSCE station 1 (Cardiovascular)**	1.25±0.50	1.67±0.52	1.33±0.58	2.25±0.50	2.00±0.00	1.50±0.58
**OSCE station 2 (Respiratory)**	2.00±0.00	2.00±0.00	2.38±0.52	2.50±0.58	2.00±0.00	2.25±0.50
**OSCE station 3 (Gastrointestinal)**	2.00±0.00	2.00±0.00	2.25±0.46	2.00±0.00	2.25±0.50	2.00±0.00
**OSCE station 4 (Urogenital)**	2.00±0.00	1.83±0.41	2.00±0.00	1.75±0.50	1.75±0.50	2.00±0.00
**OSCE station 5 (Musculoskeletal)**	2.25±0.50	2.20±0.45	2.25±0.50	2.25±0.50	2.50±0.58	2.00±0.00
**OSCE station 6 (Head & Neck)**	2.00±0.00	2.50±0.55	2.00±0.00	1.75±0.50	2.25±0.50	2.00±0.00
**OSCE station 7 (Communication)**	2.00±0.00	2.00±0.00	2.13±0.35	2.17±0.41	2.00±0.00	2.17±0.41
**Examiners Exam Difficulty （Overall）**	1.93±0.37	2.02±0.39	2.12±0.47	2.10±0.49	2.10±0.40	2.00±0.41
**Examinee Subjective Difficulty**						
**Examinees Exam Difficulty (Overall)**	1.45±0.50	1.61±0.49	1.75±0.47*	1.81±0.47*	1.54±0.51	1.60±0.49

Supplemental Table 2. Discrimination of the final scores and individual OSCE station for 6 years.

Discrimination	2018	2019	2020	2021	2023	2024
**Final score**	T=-16.55 P=0.000	T=-20.247 P=0.000	T=-20.145 P=0.000	T=-16.582 P=0.000	T=-17.826 P=0.000	T=-8.032 P=0.000
**OSCE station 1 (Cardiovascular)**	T=-3.743 P=0.000	T=-5.031 P=0.002	T=-3.262 P=0.003	T=-4.076 P=0.000	T=-6.785 P=0.000	T=-4.487 P=0.000
**OSCE station 2 (Respiratory)**	T=-2.54 P=0.013	T=-1.552 P=0.195	T=-5.862 P=0.000	T=-4.306 P=0.000	T=-4.944 P=0.000	T=-6.844 P=0.000
**OSCE station 3 (Gastrointestinal)**	T=1.799 P=0.076	T=-2.058 P=0.089	T=-6.785 P=0.000	T=-7.420 P=0.000	T=-4.275 P=0.000	T=-4.192 P=0.000
**OSCE station 4 (Urogenital)**	T=1.058 P=0.294	T=-2.785 P=0.032	T=-5.541 P=0.000	T=-5.544 P=0.000	T=-5.192 P=0.000	T=-5.552 P=0.000
**OSCE station 5 (Musculoskeletal)**	T=-0.930 P=0.356	T=-3.665 P=0.014	T=-4.420 P=0.000	T=-2.982 P=0.005	T=-4.958 P=0.000	T=-5.359 P=0.000
**OSCE station 6 (Head & Neck)**	T=-1.698 P=0.094	T=-3.359 P=0.014	T=-5.176 P=0.000	T=-1.908 P=0.063	T=-5.227 P=0.000	T=-4.132 P=0.000
**OSCE station 7 (Communication)**	T=-9.012 P=0.000	T=1.00 P=0.351	T=-2.893 P=0.000	T=-1.374 P=0.174	T=-2.400 P=0.000	T=-1.627 P=0.000
**Objective multiple choices examination**	T=-15.729 P=0.000	T=-13.477 P=0.000	T=-13.859 P=0.000	T=-12.045 P=0.000	T=-14.180 P=0.000	T=-13.516 P=0.000
